# Pancreatic solid pseudopapillary neoplasm, rare presentation in pediatric age group: two case reports

**DOI:** 10.3389/fonc.2025.1528793

**Published:** 2025-03-12

**Authors:** Khaled Alshawwa, Yumna Njoum, Hamza A. Abdul-Hafez, Sami Bannoura, Tawfiq Abukeshek, Hazem Ashhab, Omar Abu-Zaydeh

**Affiliations:** ^1^ Department of General Surgery, Al-Makassed Charitable Society Hospital, Jerusalem, Palestine; ^2^ Faculty of Medicine, Al-Quds University, Jerusalem, Palestine; ^3^ Department of Medicine, Faculty of Medicine and Health Sciences, An-Najah National University, Nablus, Palestine; ^4^ Department of Pathology, Al-Makassed Charitable Society Hospital, Jerusalem, Palestine; ^5^ Department of Radiology, Al-Makassed Charitable Society Hospital, Jerusalem, Palestine; ^6^ Department of Gastroenterology, Al-Quds University Medical School, Jerusalem, Palestine

**Keywords:** pancreatic tumor, pediatric, low-grade malignant tumor, solid pseudopapillary neoplasm, case report

## Abstract

Solid pseudopapillary neoplasm (SPN) is a rare tumor that primarily affects young females. It is typically found in the pancreas and is often asymptomatic until it grows to a large size. SPN is more frequently located in the body or tail of the pancreas in adults, whereas in children, it is more commonly found in the head of the pancreas. In this report we present two female patients in the pediatric age group who were diagnosed with pancreatic SPN, each presenting with nonspecific symptoms, one with recurrent epigastric pain and nausea, the other with carpopedal spasms. Imaging showed large, cystic-solid pancreatic masses in both. Each case underwent a Whipple procedure (pancreaticoduodenectomy) with R0 resection. Pathology confirmed SPNs without lymphovascular or perineural invasion, and all surgical margins were clear. This series underscores SPN’s varied presentations and favorable surgical outcomes in young adolescents. SPN is a rare neoplasm with low malignant potential that can present as a large abdominal mass. Although surgical resection is the treatment of choice, the optimal surgical approach remains controversial. Early detection and timely management are essential for a favorable outcome. Clinicians should consider SPN in the differential diagnosis of young females presenting with epigastric or pancreatic masses. This report highlights the importance of early detection and timely management of SPN.

## Introduction

Solid pseudopapillary neoplasms (SPNs) are rare, low-grade malignant tumors of the pancreas. They account for about 1–3% of all pancreatic tumors and are most commonly observed in young women, with a notable peak incidence in the second to third decades of life (1, 2). Although the exact cause is unclear, SPNs are thought to orient from pluripotent pancreatic cells, contributing to their unique histological and molecular profiles, which include mutations in the CTNNB1 gene (1).

Patients often present asymptomaticlly, with an upper abdominal mass discovered incidentally during imaging for unrelated reasons. When symptoms are present, they are usually nonspecific, such as mild abdominal discomfort. Jaundice is an uncommon presentation (3). The diagnosis is usually incidental, and it can be reached by computed tomography (CT) or magnetic resonance imaging (MRI) (4). Recognizing the diverse clinical presentations of SPN, understanding the morphology of its clear cell variant, and utilizing ancillary immunohistochemistry appropriately can help prevent diagnostic errors (5). Imaging plays a crucial role in making diagnosis of SPN, solid and solid-cystic masses of low echo were found in the pancreas in ultrasonic examinations. CT scan found masses of low density in the pancreas, while irregular enhancement appeared in the circumference of all tumors in enhanced CT scan sequences (6).

In this report, we present two cases of pancreatic SPN in pediatric patients, both of whom exhibited nonspecific symptoms. Each case highlights the diagnostic and management challenges associated with SPN in a younger age group, emphasizing the importance of differential diagnosis, tailored imaging studies, and surgical intervention in achieving favorable outcomes.

## Case description 1

An 11-year-old previously healthy female patient presented with a one-year history of nonspecific, dull epigastric pain radiating to the back. Initially, the pain was mild and intermittent, so she was reassured and managed symptomatically by analgesics and proton pump inhibitors (PPI), which provided partial improvement of pain. However, the pain was worsening and frequently reoccurs every few months. The patient also reported development of nausea, decrease oral intake, and loss of appetite. She denied fever, vomiting, and jaundice.

**Figure 1:**
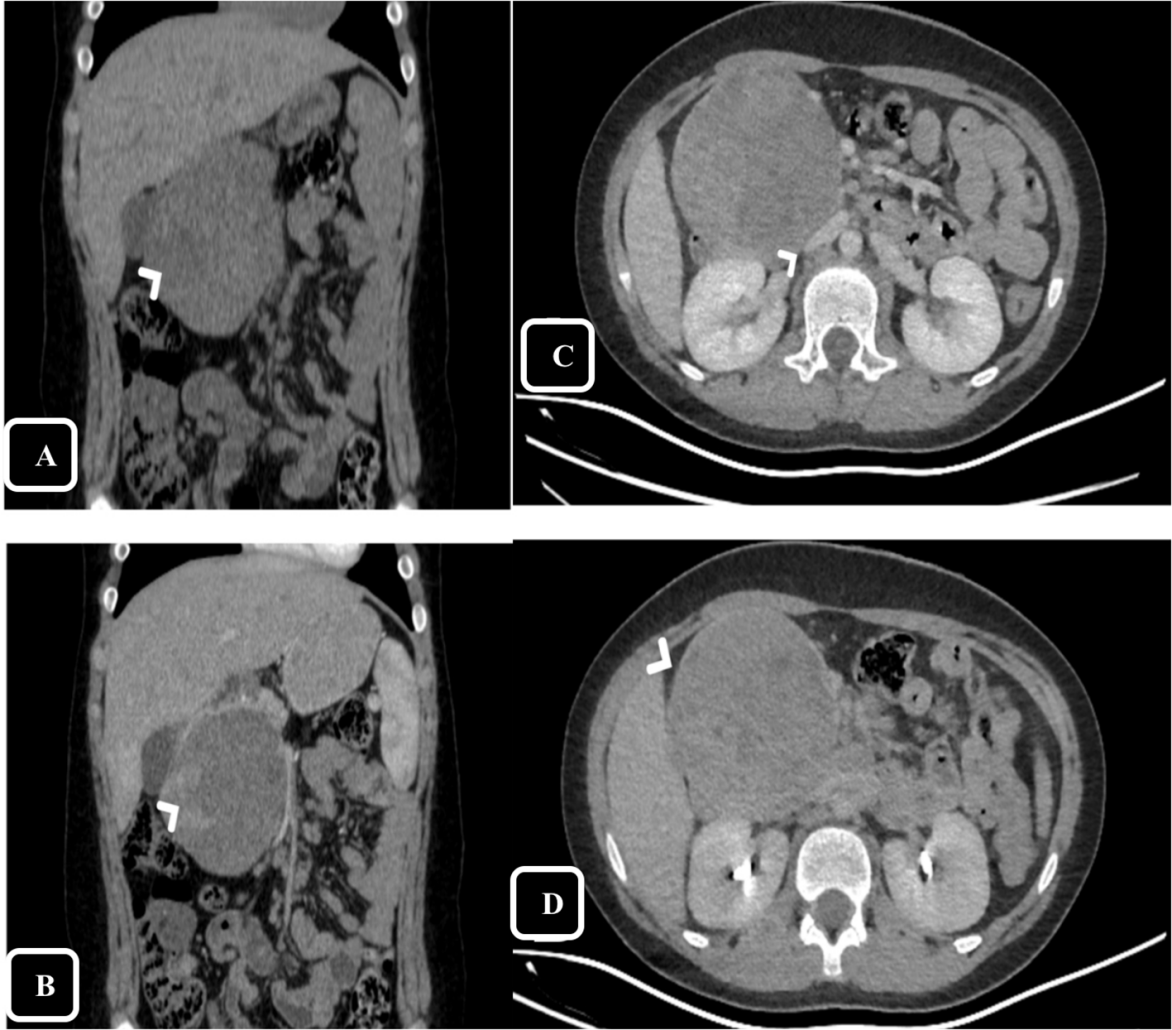
Computed Tomography (CT), coronal reformatted images on pre-contrast **(A)** and arterial phase **(B)** show large mass in the head of pancreas with claw signs of the pancreatic head appreciated on arterial phase helps defining the origin of the mass. The peripheral solid component shows enchantment on the arterial phase (arrow head). Axial reformatted images on portal **(C)** and delayed phase **(D)** show the mass effect and compression over The inferior vena cava (IVC) [arrow head in **(C)**], on delayed phase the thin enhancing rim is appreciated [arrow head in **(D)**].

Upon examination, the patient’s vital signs were within normal ranges. Abdominal examination was unremarkable except for epigastric fullness without tenderness. Abdominal ultrasound was performed and showed a heterogeneous mass measuring of 8 × 7 cm in the right upper abdomen, suspected to arise from segment I of the liver, raising concerns for hepatoblastoma. Therefore, for further evaluation, a computed tomography (CT) scan of the abdomen was conducted, showing a large, well-defined rounded mass in the head of the pancreas with both solid and cystic components, measuring 8.5 × 8 × 9.5 cm (**Figure 1**). On delayed imaging, the enhanced rim was well-seen, and there was no evidence of internal calcifications or pancreatic and biliary ductal dilation. However, mass compressive effect on adjacent structures was noted, mostly the inferior vena cava (IVC) (**Figure 1**). The patient subsequently underwent esophagogastroduodenoscopy (EGD) with endoscopic ultrasound, revealing a large heterogeneous mass extending from the head of the pancreas to the porta hepatis and liver bed. A fine needle biopsy of the mass was obtained, and histopathological analysis revealed a diagnosis of pancreatic SPN.

The case was discussed by a multidisciplinary team and recommended surgical resection. The patient underwent surgery through a Modified Makkoushi incision. Intra-operatively, a well-localized mass in the head of the pancreas mass was identified. The tumor was adherent to the duodenum, with obvious prominent, dilated cisterna chyli and small bowel lymphatic ducts. No There was no evidence of liver lesions or distant metastases. A Pancreaticoduodenectomy, also known as Whipple procedure, was performed with R0 resection of the mass, followed by Roux-en-Y reconstruction. Postoperatively, the patient had an uneventful course. Six days later, the patient was discharged home in good general condition and tolerating oral diet.

**Figure 2:**
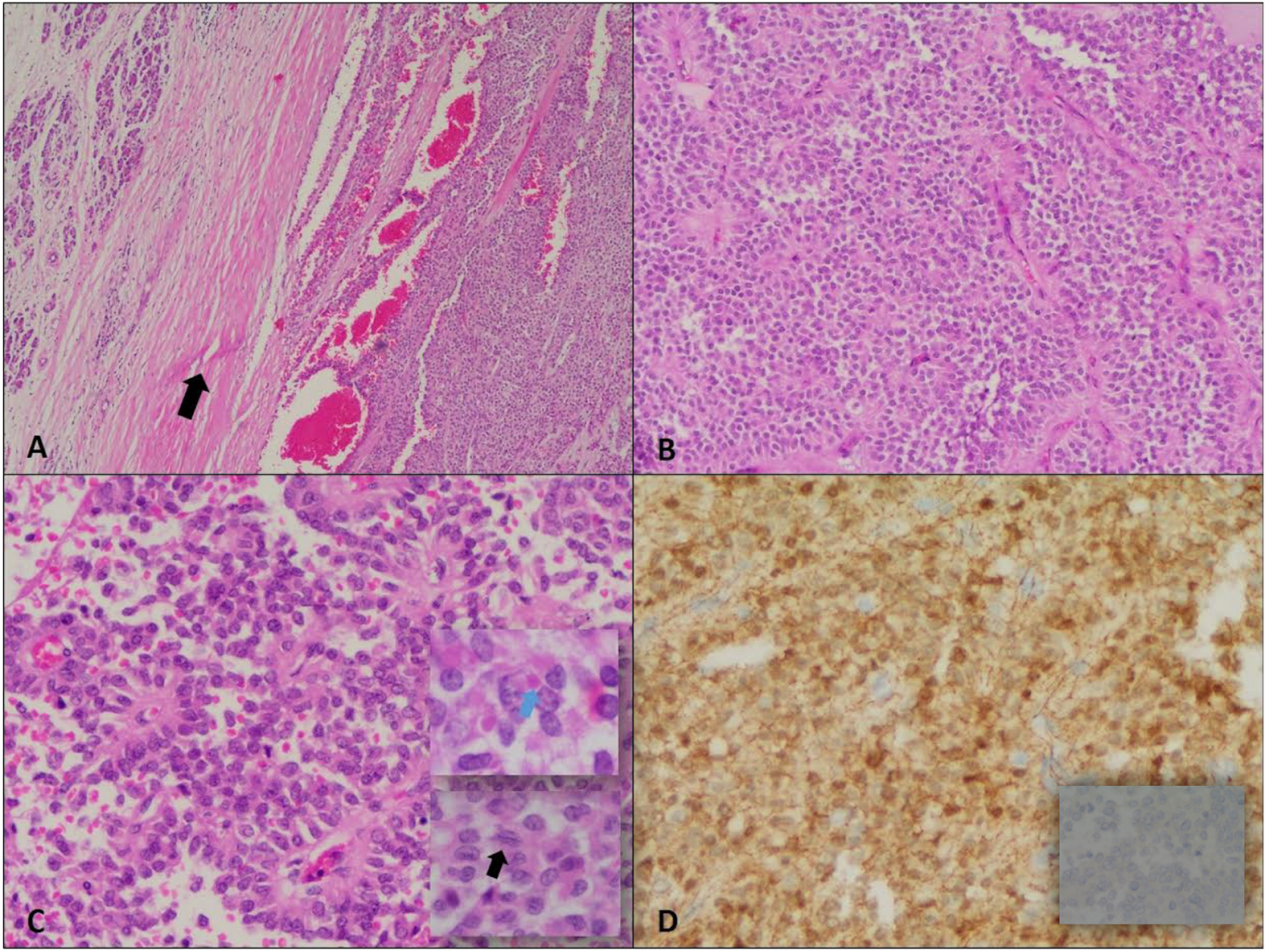
Solid pseudopapillary neoplasm. **(A)** Encapsulated neoplasm with presence of fibrous capsule (arrow) around the tumor on the right half separating it from normal pancreas on the left (H&E, 4X). **(B)** The tumor shows relatively solid areas rich in capillary-sized blood vessels (H&E, 10X). **(C)** Areas with pseudopapillary growth are noted resulting from tumor cell detachment from blood vessels (H&E, 20X); the neoplastic cells are monotonous with moderate amounts of eosinophilic cytoplasm, and presence of hyaline globules (blue arrow). The nuclei show fine chromatin distribution along with presence of longitudinal grooves (black arrow). **(D)** Positive Beta-catenin immunostain (nuclear and cytoplasmic) (20X) and negative chromogranin A immunostain (40X).

Gross pathology revealed a 9 × 9 × 8 cm tumor, and immunohistochemistry confirmed the diagnosis of SPN. There was no evidence of lymphovascular or perineural invasion, and all surgical margins were negative of tumor cells. A total of nine lymph nodes were excised, none of which showed malignancy. The final pathological stage was pT3, pN0, pMx. Immunohistochemical staining demonstrated that the tumor cells were positive for beta-catenin (both nuclear and cytoplasmic) and negative for chromogranin (**Figure 2**). Based on these findings, the oncology team recommended active surveillance without further adjuvant treatment.

## Case description 2

A 14-year-old female patient presented to our hospital with complaints of recurrent, painful carpopedal spasms for two years. Initially, the patient was found to have low calcium level, then was discharged on oral calcium with improvement of her symptoms. Two months prior to presentation, she had another attack of painful carpopedal spasm without perioral or limb numbness. She also reported a dull right-sided abdominal pain not associated with eating, so she sought medical advice in our hospital. The patient denied weight loss, jaundice, dark urine, and clay stool. The patient had an unremarkable medical history. Her surgical history included an inguinal hernia repair at the age of 8.

**Figure 3:**
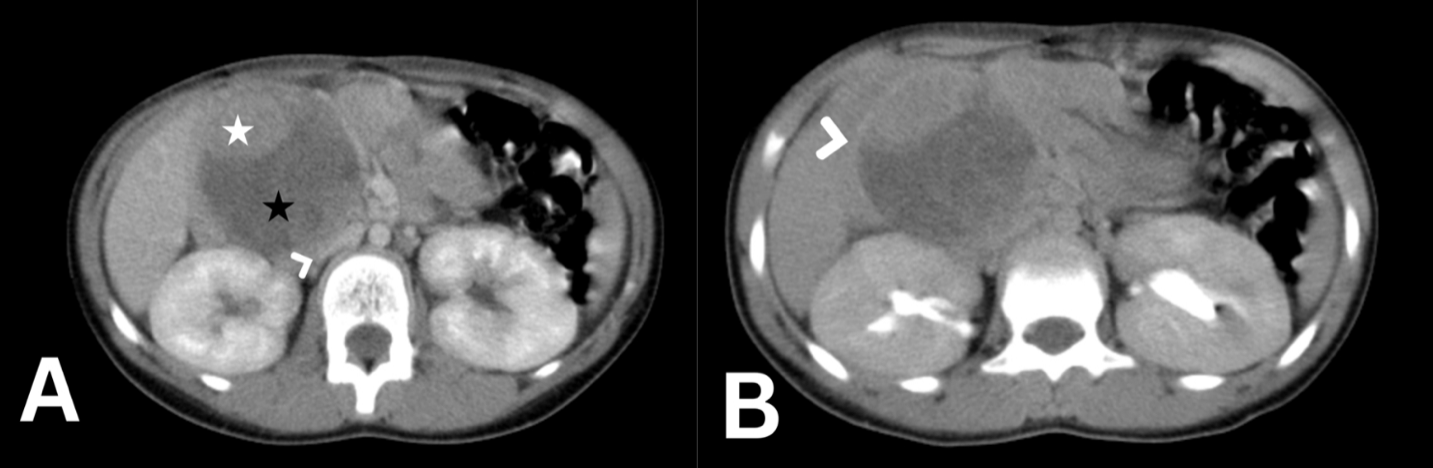
Abdomen CT scan axial cut with IV contrast in portovenous phase **(A)** showing a 7 cm rounded mass arising from the head of pancreas with solid [white star in **(A)**] and cystic (black star in A) components severely compressing the inferior vena cava [arrow head in **(A)**]. the delayed phase of the axial abdomen CT scan **(B)** shows the enhancing peripheral fibrous rim of the tumor [arrow head in **(B)**].

Upon examination, the patient’s vital signs were normal. Abdominal examination showed non-tender, palpable mass in the right abdomen, measuring about 2 × 3 cm on palpation. Laboratory tests showed in **Table 1**. An abdominal ultrasound revealed a well-defined rounded lesion in epigastric area measuring about 6 × 5.7 cm predominantly hyperechoic with cystic changes, no vascularity detected with doppler exam. For further examination, abdominal CT was performed, showing a round, well defined encapsulated mass lesion, measuring 6.7 × 6.4 × 6.2 cm, appears to have originated from the head of pancreas (**Figure 3**). The lesion is heterogeneous of both cystic and solid components, with predominant cystic portion and peripheral solid component. The lesion caused a mass effect on the adjacent duodenal loops, abutting the anterior aspect of the right kidney posteriorly. Apart from that, the pancreatic body and tail appear normal with no pancreatic duct dilation. Taking into account the patient’s age and Imaging features, Solid pseudopapillary tumor is the main differential.

**Table 1:** Laboratory findings for the two cases.

Lab test	Case 1.	Case 2.
**HGB**	11.6 g/dl	12.6 g/dl
**ALT**	9.3 U/L	14.1 U/L
**AST**	17.5 U/L	21.5 U/L
**Alkaline phosphatase**	148 U/L	353 U/L
**Total bilirubin**	0.4 mg/dl	0.21 mg/dl
**AFP**	<1	NA
**CEA**	0.6	NA
**CA19-9**	7.5	NA
**Lipase**	91 U/L	NA
**Amylase**	105 U/L	NA

HGB, Hemoglobin; ALT, Alanine Aminotransferase; AST, Aspartate Aminotransferase; AFP, Alpha-Fetoprotein; CEA, Carcinoembryonic Antigen; CA19-9, Carbohydrate Antigen 19-9; NA, Not Available.

However, EGD with endoscopic ultrasound was performed, revealing a large heterogeneous 5 × 6 cm lesion of the head of the pancreas with both cystic and solid components. Fine needle biopsy was taken and sent to histopathology. Histopathological examination showed few clusters of bland and uniform cells with moderate cytoplasm in addition to cholesterol crystals, calcification and eosinophilic hyaline globules suggestive of pseudopapillary pancreatic neoplasm.

The case was discussed by a multidisciplinary team and recommended surgical resection. The patient underwent surgery through a Kocher incision extending to midline. Intra-operatively, a well-localized, large tumor in the head of the pancreas mass was observed. Moreover, malrotation of the small bowel to the right side and paraduodenal hernia (Landzert type) with herniated small bowel through a peritoneal sac also identified during the surgery. No signs of liver lesions or distant metastasis were observed. A pancreaticoduodenectomy, also known as the Whipple procedure, was performed with resection of the mass, followed by Roux-en-Y reconstruction. Additionally, to address the paraduodenal hernia, mesocolic repair was performed. Postoperatively, the patient had an uneventful course. 10 days later, the patient was discharged home in good general condition and tolerating oral diet.

Gross pathology revealed a 7 × 6.5 × 5 cm tumor, and immunohistochemistry confirmed the diagnosis of SPN. There was no evidence of lymphovascular or perineural invasion, and all surgical margins were negative of tumor cells. A total of nine lymph nodes were excised, none of which showed malignancy. Immunohistochemical staining demonstrated that the tumor cells were positive for beta-catenin (both nuclear and cytoplasmic) and negative for chromogranin. Based on these findings, the oncology team recommended active surveillance without further adjuvant treatment.

## Discussion

Pancreatic SPN is an uncommon low-grade malignant tumor, first described by Frantz in 1959 (7). Our cases describe a rare presentation of SPN in an eleven-year-old female and fourteen-year-old patients with nonspecific epigastric pain, and recurrent, painful carpopedal spasm, respectively. The diagnosis was reached after imaging studies, including abdominal ultrasound and CT scan, followed by EGD with endoscopic ultrasound and biopsy.

The male-to-female ratio is 1:10 and the mean age at presentation is 22 years. Female SPN shows a bimodal age-frequency distribution with an early-onset incidence at 28 years and a late-onset peak incidence at 62 years, while male SPN presented a unimodal distribution with peak incidence at an approximate age of 64 years (8).

In agreement with previous studies, our cases highlight the importance of imaging in the diagnosis of SPN. Abdominal ultrasound and CT scan are commonly used showing a well-defined mass in the pancreas with both solid and cystic components, and enhancement of the peripheral solid component on both arterial and portal phases (9). EGD with endoscopic ultrasound and biopsy can provide a definitive diagnosis. MRI is the most specific imaging modality clearly identifying the soft tissue components of the lesion, perilesional invasion and its mass effect on adjacent important structures like the inferior venacava, pancreatic and biliary ducts. It typically shows mixed solid and cystic appearance, well-defined margins, peripheral capsule, and internal solid portions showing progressive enhancement.

The clinical presentation of SPN is usually nonspecific, with most patients being asymptomatic (3). Symptoms, when present, are usually mild and nonspecific, including abdominal discomfort or pain, nausea, and decreased appetite. In our cases, one of them complained of nonspecific dull aching epigastric pain radiating to the back for about a year, which recurred every few months with increasing intensity, along with nausea and decreased oral intake. The other complained of hypocalcemic symptoms, including recurrent, painful carpopedal spasms, for two years, which is unusual manifestation of pancreatic SPN.

The major differential diagnosis includes pancreatic neuroendocrine tumor (PanNET). Distant metastases are uncommon at the time of diagnosis (10). Regardless of stage, patients with SPN who underwent surgical interventions showed significantly better prognosis than those without surgical interventions. Moreover, patients with lymphatic dissection had a significantly better prognosis than those without lymphatic dissection. Male patients had significantly poorer Overall survival (OS) and disease-specific survival (DSS) compared with female patients (8). Most SPNs exhibit a benign course, still malignancy can occur in about 15% of cases, they may metastasize or invade adjacent structures. The majority of such tumors are located in the pancreatic body and tail in adults, while more frequent in the head of the pancreas in children (11).

In a study of lymph node metastasis in pancreatic cancer, Malleo et al. found that 88.2% of 424 patients undergoing surgery had cancerous lymph nodes. Critically, the location and the numbers of these nodes proved prognostically significant. Even minimal involvement of nodes further from the tumor (second echelon) was a red flag. This has important implications for surgical and pathological practice, suggesting that a thorough examination of all regional nodes, not just those immediately adjacent to the tumor, is crucial for accurate staging and personalized treatment planning (12).

Surgical resection remains the treatment of choice for SPN, offering a favorable prognosis. Both of our cases confirm that, regardless of stage, patients who undergo surgical intervention demonstrate significantly better prognoses than those who do not. Moreover, patients with lymphatic dissection had a significantly better prognosis than those without lymphatic dissection. Female patients had a significantly better Overall survival (OS) and disease-specific survival (DSS) compared with male patients. Both of our cases, the patients underwent an uneventful surgical intervention, and the tumor was resected successfully (13).

In conclusion, SPN is a rare pancreatic tumor that usually presents in young women. These cases highlight the rare presentation of SPN in an 11-year-old female with nonspecific epigastric pain and nausea and a 14-year-old female with hypocalcemic symptoms, including carpopedal spasms. Imaging studies, including abdominal ultrasound and CT scan, followed by EGD with endoscopic ultrasound and biopsy, can provide a definitive diagnosis. Surgical resection remains the standard treatment and is associated with excellent prognosis. Early diagnosis and appropriate management are crucial to achieve optimal outcomes in patients with SPN.

## Data Availability

The raw data supporting the conclusions of this article will be made available by the authors, without undue reservation.
